# A genomic assessment of species boundaries and hybridization in a group of highly polymorphic anoles (*distichus* species complex)

**DOI:** 10.1002/ece3.2751

**Published:** 2017-04-15

**Authors:** Daniel J. MacGuigan, Anthony J. Geneva, Richard E. Glor

**Affiliations:** ^1^ Department of Ecology and Evolutionary BiologyYale UniversityNew HavenCTUSA; ^2^ Department of Organismic and Evolutionary BiologyHarvard UniversityCambridgeMAUSA; ^3^ Herpetology DivisionBiodiversity InstituteUniversity of KansasLawrenceKSUSA; ^4^ Department of Ecology and Evolutionary BiologyUniversity of KansasLawrenceKSUSA

**Keywords:** AFLP, *Anolis*, biogeography, dewlap, *distichus*, species delimitation

## Abstract

Delimiting young species is one of the great challenges of systematic biology, particularly when the species in question exhibit little morphological divergence. *Anolis distichus*, a trunk anole with more than a dozen subspecies that are defined primarily by dewlap color, may actually represent several independent evolutionary lineages. To test this, we utilized amplified fragment length polymorphisms (AFLP) genome scans and genetic clustering analyses in conjunction with a coalescent‐based species delimitation method. We examined a geographically widespread set of samples and two heavily sampled hybrid zones. We find that genetic divergence is associated with a major biogeographic barrier, the Hispaniolan paleo‐island boundary, but not with dewlap color. Additionally, we find support for hypotheses regarding colonization of two Hispaniolan satellite islands and the Bahamas from mainland Hispaniola. Our results show that *A. distichus* is composed of seven distinct evolutionary lineages still experiencing a limited degree of gene flow. We suggest that *A. distichus* merits taxonomic revision, but that dewlap color cannot be relied upon as the primary diagnostic character.

## 1 Introduction

The formation of new species is typically a gradual process that occurs over thousands or even millions of generations. As this makes speciation difficult to observe experimentally, investigating how and why speciation occurs tends to rely heavily on observations of species at varying stages of the speciation process—the snapshot approach to studying speciation. Relatively young species are particularly important but also the hardest to identify because they often fail to meet one or more of the criteria expected of deeply divergent species. They may, for example, exhibit incomplete reproductive isolation, readily hybridize with other species, or be difficult to distinguish morphologically or genetically (Coyne & Orr, 2004; Knowles & Carstens, 2007; Maddison & Knowles, 2006; Shaffer & Thomson, 2007). Speciation is a continuum under the general lineage concept, and criteria are expected to accumulate gradually (de Queiroz, 2007).

Our goal here is to use genomic data to identify candidate species within a polytypic lizard species that may include a number of young lineages at varying stages of divergence (Geneva, Hilton, Noll & Glor, 2015; Glor & Laport, 2012; Ng & Glor, 2011). The Hispanolian bark anole (*Anolis distichus*) is a trunk‐dwelling lizard species that currently includes more than a dozen subspecies distributed across Hispaniola and the Bahamas (Schwartz, 1968). These subspecies are primarily delimited by differences in the color and pattern of their dewlaps, throatfans that are extended by males during behavioral displays (Schwartz, 1968). Dewlaps in *A. distichus* range from pale yellow to wine red, with many variants in between; most dewlap color and pattern variation occurs among geographically circumscribed populations, but considerable variation can also exist within some populations (Lambert, Geneva, Mahler & Glor, 2013; Schwartz, 1968). Because the dewlap is thought to play a critical role in species recognition and sexual selection, dewlap divergence has been used as a proxy for reproductive isolation and is often used to delimit species boundaries (e.g. Lotzkat, Bienentreu, Hertz & Köhler, 2011; Poe & Yañez‐Miranda, 2008; Velasco & Hurtado‐Gómez, 2014). However, in the case of *A. distichus* and a few other polymorphic anole species, populations with strikingly different dewlaps have been recognized as subspecies or unnamed geographic populations rather than distinct species because they appear to hybridize where they come into contact (Heatwole, 1976; Schwartz, 1968; Schwartz, 1974; Underwood & Williams, 1959). This decision to recognize dewlap color variation at the subspecific level (or not at all) is supported by more recent evidence that dewlap color and pattern variation in *A. d. distichus* may represent an adaptive response to local signaling conditions rather than an indicator of reproductive isolation (Ng, Kelly, MacGuigan & Glor, 2013; Ng, Landeen, Logsdon & Glor, 2012; Webster, 1977).

Prior molecular genetic studies of *A. distichus* provided mixed support for the evolutionary independence of the subspecies diagnosed by differences in dewlap color and pattern. Early allozyme studies revealed molecular differentiation and reduced gene flow at the contact zone between some subspecies (Case & Williams, 1984) but not others (Case, 1990; Case & Williams, 1984; Williams, 1977; Williams & Case, 1986). Meanwhile, mitochondrial DNA (mtDNA) sequence data suggested that each of the subspecies found in the Dominican Republic form distinct and deeply divergent clades, with the exception of the widespread subspecies *A. d. dominicensis*, which is associated with multiple mtDNA clades (Glor & Laport, 2012). Fine‐scale studies of contact zones between pairs of subspecies involving phenotypic, mitochondrial, and microsatellite data have uncovered evidence for abrupt phenotypic and genetic divergence along narrow hybrid zones, but also evidence for extensive introgression and relatively shallow genetic differentiation (Ng & Glor, 2011; Ng, Ossip‐Klein & Glor, 2016). Multilocus phylogenetic analyses have found that while most subspecies of *A. distichus* are genetically distinct, these differences were mostly restricted to mtDNA, and several subspecies were not monophyletic (Geneva et al., 2015).

**Figure 1:**
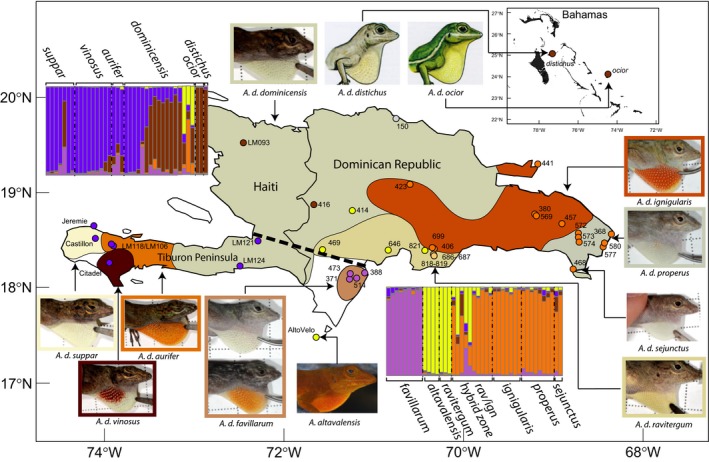
Distributions of *A. distichus* subspecies, sampling for Set 1, and results from genotypic clustering analyses conducted in STRUCTURE. Each column on the bar plots represents an individual sample. Different colors correspond to different genetic clusters. Shading of each column represents the proportion of the genome for that individual assigned to one of the genetic clusters identified by STRUCTURE. Each point on the map is a locality included in Set 1, labeled with corresponding locality numbers. The color of each locality reflects the genetic cluster to which the majority of the genomes at that locality were assigned. Locality 150 is colored gray because the genome of the specimen at that locality was not assigned to a single genetic cluster. Localities 686, 687, 818, and 819 are colored light orange to represent their admixed status. The colored regions on the map represent approximate subspecies ranges, with white space where no subspecies is present. Subspecies ranges are based on the maps of Ng et al. (2013) and Schwartz (1968). The dashed black line represents Merten's line, the boundary between Hispaniola's North and South paleo‐islands

The multilocus phylogeny of Geneva et al. (2015) also suggested for the first time that geography may be more important than dewlap color variation for delimitation of *Anolis* species. Modern Hispaniola formed when a North and a South paleo‐island merged approximately 15 mya (Graham, 2003; Iturralde‐Vinent & MacPhee, 1999). The boundary between these paleo‐islands, also known as Mertens’ Line, has long been recognized as one of the most important biogeographic boundaries on Hispaniola (Schwartz, 1980). The current boundary between the paleo‐islands (Figure 1, black dashed line) has likely remained a biogeographic barrier since the merger because it coincides with a lowlying xeric valley that is periodically inundated with seawater, and is relatively inhospitable to lizards adapted to the more mesic environments flanking the valley (Glor & Warren, 2011; Townsend, Rimmer, Latta & Lovette, 2007). *Anolis distichus* populations appear to have diverged across Mertens’ line, with the deepest phylogenetic split dividing clades of subspecies found primarily on either the North or the South paleo‐island (Geneva et al., 2015).

In spite of this prior work, no study of *A. distichus* has involved range‐wide assessment of genomic variation with the goal of identifying candidate species. Utilizing amplified fragment length polymorphism (AFLP) genome scans, we apply a two‐step process of candidate species discovery and validation (Carstens, Pelletier, Reid & Satler, 2013). We specifically test whether the *A. distichus* subspecies delimited by dewlap color and pattern correspond with genetically distinct populations that may warrant recognition as distinct species under the general lineage species concept (de Queiroz, 2007). Additionally, we test whether divergence across Mertens’ line occurred in the *A. distichus* complex and contributed to the group's current taxonomic diversity. We then use AFLP genome scans on a finer geographic scale to test Ng et al.'s (2016) prediction that two pairs of subspecies characterized by different dewlap color are genetically distinct and experiencing limited gene flow where they come into contact. With our genomic perspective, we also test the hypothesis that dewlap color has diverged repeatedly within and among populations of *A. distichus*. Our results shed light on the species‐level diversity within *A. distichus*, the efficacy of dewlap color as a diagnostic character, and the role of biogeography in shaping genomic divergence.

## 2 Methods

### 2.1 Tissue sampling and DNA extraction

We obtained tissue samples for 245 lizards from 76 localities on Hispaniola and the Bahamas, representing both species in the *distichus* species complex (*A. distichus* and *A. altavalensis*) and 11 of 18 *A. distichus* subspecies. We divided these samples into three sets. The first set was designed to broadly diagnose genetically distinct populations and candidate species across the *distichus* species complex. This set initially included 92 samples from 39 localities, with broad taxonomic coverage of the *A. distichus* species complex, including *A. altavalensis* (endemic to the Hispaniolan satellite island of Alto Velo) and subspecific sampling of *A. distichus* that included all mainland Hispaniolan subspecies, two of the five Bahamian subspecies, and one of the four subspecies endemic to Hispaniolan satellite islands (Table 1, Set 1).

**Table 1:** Sampling for this study. This table includes only those individuals that passed our preliminary quality control screening

Taxon	Distribution	Localities	Individuals
Set 1: General Sampling (8 primer pairs, 534 loci, 66.75 loci/primer pair)			
*A. altavalensis*	Isla Alto Velo	1	4
*A. d. ocior*	Bahamas	1	2
*A. d. distichus*	Bahamas	1	1
*A. d. aurifer*	Hispaniola; South Paleo‐island; Tiburon Peninsula	2	3
*A. d. vinosus*	Hispaniola; South Paleo‐island; Tiburon Peninsula	1	9
*A. d. suppar*	Hispaniola; South Paleo‐island; Tiburon Peninsula	2	7
*A. d. favillarum*	Hispaniola; South Paleo‐island; Barahona Peninsula	4	9
*A. d. dominicensis*	Hispaniola; North and South Paleo‐islands	7	17
*A. d. properus*	Hispaniola; North Paleo‐island; Western Dominican Republic	5	8
*A. d. ignigularis*	Hispaniola; North Paleo‐island; Central Dominican Republic	9	13
*A. d. ravitergum*	Hispaniola; North Paleo‐island; South‐central Dominican Republic	3	3
*A. d. ignigularis/ravitergum*	Hispaniola; North Paleo‐island; South‐central Dominican Republic	2	4
*A. d. sejunctus*	Hispaniola; North Paleo‐island; Isla Soana	1	2
Set 2: *A. d. ignigularis*/*A. d. ravitergum* hybrid zone (6 primer pairs, 552 loci, 92 loci/primer pair)			
*A. d. ignigularis*	Hispaniola; North Paleo‐island; South‐central Dominican Republic	1	13
*A. d. ravitergum*	Hispaniola; North Paleo‐island; South‐central Dominican Republic	1	14
*A. d. ignigularis/ravitergum*	Hispaniola; North Paleo‐island; South‐central Dominican Republic	21	50
Set 3: *A. d. dominicensis*/*A. d. ignigularis* hybrid zone (6 primer pairs, 836 loci, 139.33 loci/primer pair)			
*A. d. dominicensis*	Hispaniola; North Paleo‐island; Samaná Peninsula	7	23
*A. d. ignigularis*	Hispaniola; North Paleo‐island; Samaná Peninsula	4	18
*A. d. dominicensis/ignigularis*	Hispaniola; North Paleo‐island; Samaná Peninsula	2	10

Our second set of 92 individuals was designed to assess genetic divergence and hybridization between *A. d. ignigularis* and *A. d. ravitergum* across a hybrid zone along the Baní River in the south‐central Dominican Republic. Previous work has suggested abrupt phenotypic and genetic differentiation across a narrow hybrid zone (<5 km), with limited evidence for widespread gene flow or introgression (Ng & Glor, 2011; Ng et al., 2016; Table 1, Set 2).

Our third set of 59 samples was designed to assess genetic divergence and hybridization between *A. d. ignigularis* and *A. d. dominicensis* across a hybrid zone at the base of Samaná Peninsula in the northeastern Dominican Republic. Previous work along this zone indicates abrupt phenotypic and genetic divergence, albeit with considerably shallower genetic differentiation than the transect between *A. d. ignigularis* and *A. d. ravitergum* (Ng & Glor, 2011; Ng et al., 2016; Table 1, Set 3).

We extracted DNA from tail tips or liver samples stored in 95% ethanol at −80°C using either a Wizard SV Genomic DNA Purification System kit (Promega Corp.) or via a phenol chloroform extraction protocol modified from Laird et al. (1991). For phenol chloroform extractions, we combined up to 20 ng of tissue with 250 μl of TENSII (base solution 4 ml 5 mol/L NaCl, 50ml 1 mol/L Tris pH 8, 2 ml 0.5 mol/L EDTA pH 8, 844 ml H${}_{2}$O, 100 ml 10% SDS), 20 μl of proteinase K (20 μg/μl), and 5μl RNase A solution before incubating for 16–18 hr at 55°C. Following incubation, we transferred this solution to a prespun (15,000 *g* for 1–2 min) Phase Lock Gel^TM^ (PLG) 2 ml heavy tube (5 Prime, Inc), added 0.5 ml of phenol:chloroform:isoamyl alcohol (PCI, 25:24:1), and mixed via repeated inversion. We then centrifuged at 14,000 *g* in an Eppendorf model 5,424 microcentrifuge for 5 min before transferring the resultant aqueous phase to a fresh prespun PLG 2 ml tube heavy tube. We next added 0.5 ml of chloroform:isoamyl alcohol (CI, 24:1) to the sample in the PLG2 ml tube, mixed by repeated inversion, and centrifuged the tube at 14,000 *g* for 5 min before transferring the resultant aqueous phase to a fresh microcentrifuge tube. We then added 30 ml of sodium acetate 3 mol/L, pH 5.2) and 1.25 ml of 95% ethanol before incubating at −20°C overnight. Finally, we centrifuged this mixture at 14,000 *g* for 20 min, rinsed with 1 ml of 95% ethanol, centrifuged again at 14,000 *g* for 10 min, and ultimately re‐suspended the resulting DNA pellet in 200 μl H${}_{2}$O.

### 2.2 Molecular methods

#### 2.2.1 AFLP genotyping

AFLPs can provide a large amount of genomic data to address questions about population structure and hybridization (Bensch & Akesson, 2005; Mueller & Wolfenbarger, 1999) and are more cost‐effective than other methods for acquiring genomic data (e.g., GBS or similar SNP‐based approaches). Because AFLPs are dominant markers, they generally suffer from reduced information content relative to sequencing or SNP‐based approaches (Elshire et al., 2011). AFLPs may also suffer greatly from genotyping error (Crawford, Koscinski & Keyghobadi, 2012), but these errors are accounted for by the methods outlined below.

AFLP genotyping involved four steps: (1) digestion of genomic DNA and ligation of adaptors, (2) preselective PCR amplification of genomic DNA, (3) selective PCR amplification with fluorescently labeled primers, and (4) scoring of fragments resulting from selective amplification. We followed Lambert et al. (2013) in using AFLP protocols modified from Vos et al. (1995). To begin, we digested 200 ng of genomic DNA per sample using two restriction enzymes, *Eco*R1 and *Mse*1, with each restriction digestion reaction having a total volume of 20 μl and consisting of 17.6 μl of DNA suspended in H${}_{2}$O, 2.0 μl of New England Biolabs (NEB) 10X CutSmart Buffer, 0.25 μl NEB *Eco*R1 (20 units/μl), 0.05 μl NEB *Mse*1 (10 units/μl), and 0.1 μl NEB 100X BSA (20 mg/ml). The reaction conditions for digestion were 37°C for 180 min followed by 60°C for 15 min. We immediately followed digestion with ligation of custom adaptors to the sticky ends of restriction fragments. Each ligation reaction included the digested product, 5.8 μl H${}_{2}$O, 2 μl 10X T4 ligase buffer (with ATP), 0.2 μl T4 DNA ligase (400 units/μl), 1 μl *Eco*R1 adaptor (5 μmol/L), and 1 μl *Mse*1 adaptor (50 μmol/L). Ligation product was incubated overnight at 37°C. We ran ligation product on a 1.5% agarose gel to check whether samples had successfully undergone the first step. If digestion and ligation were successful, we observed a diffuse smear (or sometimes distinct bands) between 200 and 1,000 basepairs (bp). Samples that failed the digestion and ligation step were rerun until successful or were excluded from the final dataset.

We performed one round of preselective PCR amplification using primers complementary to the adaptor sequence, but with one additional nucleotide (adenine for *Eco*R1 adaptors, cytosine for *Mse*1 adaptors) at the 3’ end of the primer. Each preselective PCR amplification reaction included 22.8 μl H${}_{2}$O, 5 μl *Eco*R1 primer (10 μmol/L), 5 μl *Mse*1 primer (10 μmol/L), 5 μl MgSO${}_{4}$, 5 μl 10X BioBasic Buffer, 5 μl dNTPs (0.5 mmol/L), 0.25 μl of Taq polymerase, and 2 μl of the ligation product. The reaction conditions for preselective amplification were 94°C for 2 min followed by 26 cycles of 94°C for 1 min, 56°C for 1 min, and 72°C for 1 min, with a final step of 72°C for 1 min. We ran preselective amplification product on a 1.5% agarose gel to check if samples had successfully amplified. If preselective amplification was successful, we observed a smear or distinct bands between 50 and 500 bp. Samples that failed the preselective amplification were rerun until successful or excluded from the final dataset.

Following the preselective amplification, we performed selective PCR amplification on products of the preselective amplification using primers identical to the preselective primers, but with the addition of two nucleotides at the 3’ end. We used a total of six primers for selective amplification, including two primers complementary to the *Eco*R1 adaptor sequence and three primers complementary to the *Mse*1 adaptor sequence. For each selective PCR amplification, we used 11.4 μl H${}_{2}$O, 2.5 μl 10x BioBasic buffer, 2.5 μl dNTPs (0.5 mmol/L), 2.5 μl *Mse*1 selective primer (2 μmol/L), 2.5 μl *Eco*R1 labeled selective primer (2 μmol/L), 2.5 μl MgSO${}_{4}$, 0.125 μl Taq polymerase, and 1 μl of preselective amplification product. Selective amplification reaction conditions were 94°C for 1 min, 12 cycles of 94°C for 30 s, 65°C for 30 s (decreased by 1°C per cycle), and 72°C for 1 min, 23 cycles of 94°C for 30 s, 56°C for 30 s, and 72°C for 1 min, with a final step of 72°C for 1 min.

In the selective amplification step, we utilized two *Eco*R1 florescent primers (one labeled with VIC and one labeled with 6‐FAM) and three *Mse*1 primers for a total of total of six unique primer pair combinations. For our first set of samples, we used a fourth *Mse*1 primer for a total of eight unique primer pairs to increase the number of loci (Table  1). All fragment analyses were performed by the Functional Genomics Center at the University of Rochester Medical Center using an Applied Biosystems 3730 Genetic Analyzer with a LIZ500 size standard.

#### 2.2.2 AFLP scoring and error analysis

We individually analyzed every primer pair for each set of samples, as well as for a concatenated dataset containing all samples. For the combined dataset, only the first six primer pairs were used. We first visually inspected and analyzed AFLP electropherograms using PeakScanner v1.0 (Applied Biosystems) with light peak smoothing and default settings. We analyzed results from PeakScanner using a modified version of the R package RawGeno (Arrigo, Tuszynski, Ehrich, Gerdes & Alvarez, 2009). For analyses of individual sets, we set the maximum bin width to two base pairs (bp), minimum bin width to one bp, minimum fragment size to 50 bp, and maximum fragment size to the observed maximum fragment length. To remove low‐intensity peaks, we set the minimum peak height threshold to 100 relative florescence units (RFU). For analyses of the concatenated dataset, we used the same settings except for maximum fragment size, which we set equal to the smallest of the observed maximum fragment sizes from the individually analyzed sets.

We used the “visualize samples” tool in RawGeno (Arrigo et al., 2009) to identify samples that had fewer AFLP peaks than expected, potentially indicating a methodological or analytical failure. The “visualize samples” tool produces a binary matrix of AFLP loci, with an AFLP peak either present or absent at a particular fragment size (Figure S1). If a sample failed in any of the marker preparation steps, it would have very few strong AFLP peaks and the “visualize samples” tool would call most loci as “absent.” For a given sample, we identified primer pairs as problematic if they had few “present” loci compared to the rest of the samples. We then removed all problematic primer pairs for that sample from the final dataset. If this procedure resulted in diagnosis of three or more problematic primer pairs for a particular sample, that sample was completely removed from the dataset prior to downstream genetic clustering and species delimitation analyses.

We exported raw peak height output data from RawGeno and used a custom R script to convert this output into a format accepted by the R package AFLPScore (Whitlock, Hipperson, Mannarelli, Butlin & Burke, 2008). We used ALFPScore to assess the mismatch genotyping error rate for duplicate samples (Whitlock et al., 2008). We duplicated 16 samples, representing 12.2% of our dataset and exceeding the recommended 5–10% (Bonin et al., 2004). To generate duplicate samples, we repeated selective amplification for eight samples from Set 1, 14 samples from Set 2, and eight samples from Set 3 (30 total samples). All duplicate samples were randomly selected. We scored AFLPs for these duplicate samples following the same protocol discussed above. We analyzed error rates for each primer pair at phenotype threshold values of 100, 250, 500, 750, and 1,000 (the minimum RFU required to call the phenotype at a specific locus as present) and at locus threshold values of 100, 250, 500, and 750 (the minimum RFU required to call a peak as a locus). We selected and applied the least strict threshold values that produced an error rate <0.05 as suggested by Zhang and Hare (2012). Our acceptable error rate is consistent with the 6–18% error rates reported for optimized phylogenetic resolution (Holland, Clarke & Meudt, 2008) and only slightly higher than the 2–5% error rates reported in several other studies using semi‐automated AFLP scoring (Bonin et al., 2004). Although mismatch error rates are difficult to compare between studies (Holland et al., 2008), our semi‐strict filtering has been demonstrated to strike an effective balance for population level studies, maximizing the number of loci recovered while minimizing the effect of background noise on genotype calling (Lambert et al., 2013).

### 2.3 Species delimitation & species tree inference

We used two methods to infer boundaries between candidate species from the AFLP data acquired for Set 1, which included broad geographic and taxonomic sampling. The first method was largely exploratory and relied on the clustering algorithms implemented in the program STRUCTURE (Pritchard, Stephens & Donnelly, 2000) to ask whether some populations or sets of populations correspond with genotypic clusters that may represent distinct species. The second method used Bayes factors and a coalescent‐based framework to quantitatively evaluate and compare a set of alternative species delimitation scenarios derived *a priori* from taxonomy, biogeography, or the genotypic clusters identified by STRUCTURE (Leaché, Fujita, Minin & Bouckaert, 2014). In addition to identifying species boundaries, we also used our AFLP data to infer a species tree that reflects evolutionary relationships between candidate species.

#### 2.3.1 Genotypic clustering analyses

To determine whether genotypically distinct populations exist in the *A. distichus* species group, we conducted genotypic clustering analyses with our Set 1 AFLP data using the program STRUCTURE (Pritchard et al., 2000). STRUCTURE uses a Bayesian Markov chain Monte Carlo (MCMC) algorithm to probabilistically assign individuals to genetic clusters (Pritchard et al., 2000). We also performed the same STRUCTURE analyses independently on the AFLP data from Set 2 and Set 3 to examine the degree of admixture along our two hybrid zone transects.

Strong confounding effects prevented us from combining all three sample sets for one STRUCTURE analysis (see Section 3). For each STRUCTURE analysis, we utilized the admixture and correlated allele frequency models. Because AFLP markers are dominant, we also employed the recessive alleles model. We ran each analysis for 1,000,000 generations, excluding 100,000 generation as burn‐in. We then used the Δ*K* method in Structure Harvester to determine the optimal number of genetic clusters (*K*) (Earl & vonHoldt, 2012; Evanno, Regnaut & Goudet, 2005). The Δ*K* method examines the rate of change in lnP(D), where lnP(D) is an estimate of the posterior probability for *K* genetic clusters. The optimal number of genetic clusters is identified as the breakpoint where the slope of lnP(D) vs. *K* begins to plateau (Evanno et al., 2005).

The Δ*K* method alone can underestimate the actual number of genetic clusters in a dataset (Evanno et al., 2005), so we employed a hierarchical version of the Δ*K* method (Coulon et al., 2008). We assigned individuals to genetic clusters based on majority (>0.5) inferred ancestry. Individuals that did not have the majority of their genotypes assigned to one cluster were excluded from subsequent hierarchical analyses, but were included in the final nonhierarchical analyses at a fixed value of *K*. Individuals were divided into subsets based on their majority cluster assignment and subjected to another round of STRUCTURE analyses using the Δ*K* method. For each round of analyses, we performed 10 independent replicate STRUCTURE runs at each value of *K*, with *K* values ranging from 1 to the maximum number of subspecies included in the dataset plus two. We set the maximum *K* to a value larger than the number of subspecies represented in each analysis to avoid forcing individuals into inappropriately few clusters (Kalinowski, 2011).

Adapting the general guidelines outlined by Coulon et al. (2008), we employed this hierarchical Δ*K* method until *K* = 1 had the highest posterior probability, <5 individuals were assigned to a genetic cluster, or no individuals had the majority of their genotypes assigned to any cluster. When no further population subdivision was possible, we calculated the total number of clusters identified during the hierarchical analyses. To generate our final clustering results, we performed 100 replicate STRUCTURE runs on the complete Set 1 dataset with *K* fixed at the total number of clusters identified by the hierarchical analyses.

#### 2.3.2 Bayes factor delimitation

**Figure 2:**
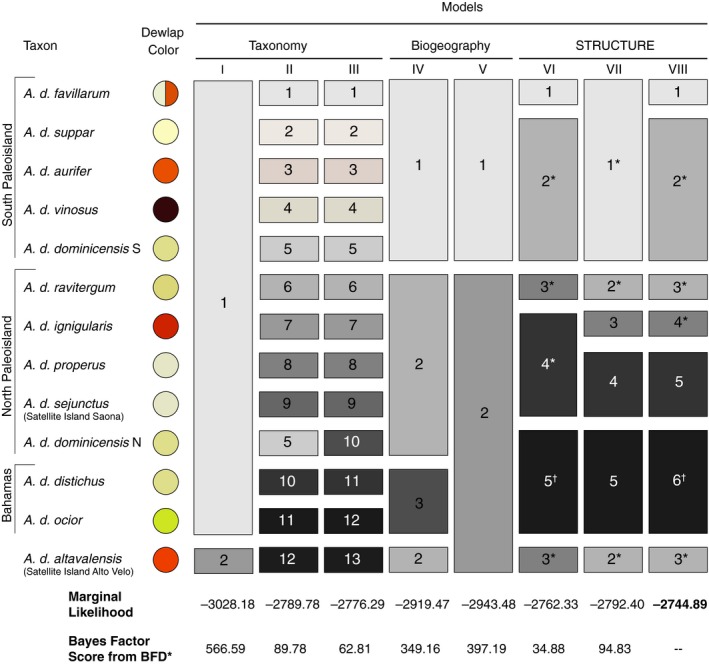
Species delimitation models along with Bayes Factor scores from BFD* analyses. Species delimitation models (roman numerals) are displayed as columns with candidate species (numbered boxes) comprised of different combinations of subspecies (rows). The subspecies *A. d. dominicensis* is split into North (N) and South (S) paleo‐island populations. Asterisks next to candidate species numbers indicate that one or more individuals from *A. d. dominicensis* N are included in the candidate species, and crosses indicate that one *A. d. suppar* individual was included in the candidate species. Marginal likelihood estimates and Bayes factor scores are noted for each species delimitation model. All Bayes factors were calculated relative to model VIII

We tested alternative species delimitation scenarios using the coalescent‐based model comparison framework outlined by Leaché et al. (2014). We generated eight species delimitation scenarios that consisted of between two and thirteen species based on (1) current taxonomy, (2) biogeography, and (3) genotypic clusters identified by STRUCTURE (Figure 2). The three models based on the current taxonomy were (I) two species corresponding to the two currently recognized species (*A. altavalensis* and *A. distichus*), (II) twelve species corresponding to *A. altavalensis* and each sampled subspecies of *A. distichus*, and (III) thirteen species including the twelve species of model II plus distinct *A. d. dominicensis* species on the North and South Hispaniolan paleo‐islands, as implied by recent multilocus phylogenetic analyses (Geneva et al., 2015). The two biogeographic models were (IV) three species, corresponding with Hispaniola's North paleo‐island (including both Hispaniolan satellite island populations as implied by the phylogeny of Geneva et al. (2015)), Hispaniola's South paleo‐island, and the Bahamas and (V) two species corresponding with Hispaniola's South paleo‐island and Hispaniola's North paleo‐island plus the Bahamas and the two Hispaniolan satellite islands. The three models based on STRUCTURE results were (VI) five species with individuals assigned based on results of the final round of STRUCTURE analyses fixed at *K* = 5 (Figure 1), (VII) five species with individuals assigned based on results of the hierarchical STRUCTURE analyses (Figure S2), and (VIII) six species with individuals assigned based on both STRUCTURE results (i.e., model VI except with *A. d. properus/sejunctus* as a separate species from *A. d. ignigularis*, as in model VII).

We assessed the relative fit of each species delimitation model using Bayes factor delimitation with genomic data (BFD*), a recently developed species delimitation method for analysis of biallelic genomic data (Leaché et al., 2014). BFD* combines the likelihood algorithm in the BEAST v.2.1.3 add‐on SNAPP v.1.1.6 (Bouckaert et al., 2014; Bryant, Bouckaert, Felsenstein, Rosenberg & RoyChoudhury, 2012) with path sampling, a method to estimate marginal likelihoods for use in Bayes factor model selection (Leaché et al., 2014). BFD* allows direct comparison of competing species delimitation models without requiring them to be nested. Following Leaché et al. (2014), we conducted path sampling with 48 steps (100,000 MCMC steps, 10,000 preburnin steps, estimating the mutation rates u and v) to estimate marginal likelihoods for each model. We used a gamma prior on the ancestral population sizes, with a shape parameter (α) of 11.75 and a scale parameter (β) of 109.73. We used a Yule prior for the species tree height and branch lengths with a lambda parameter of 0.00765. We found that use of the dominant alleles model in SNAPP dramatically increased run‐times and made species tree estimation with even a modest number of individuals and loci computationally unfeasible. The program's authors have also reported that use of this model can increase run‐times without significantly altering the results (http://beast2.cs.auckland.ac.nz/SNAPPv1.2.pdf). Therefore, we did not employ the dominant alleles model for our analyses.

For each species delimitation model, we calculated Bayes factors (BF) by dividing the marginal likelihood of the best fitting model (i.e., the model with the highest marginal likelihood) by the marginal likelihood of each competing model. We then calculated BF model selection statistics as 2 × ln(BF) (Kass & Raftery, 1995). Thus, our BF model selection statistics indicated the degree of support for the best fitting model relative to each alternative model. A BF model selection statistic between 0 and 2 reflects weak support, between 2 and 6 reflects positive support, between 6 and 10 reflects strong support, and greater than 10 reflects decisive support (Kass & Raftery, 1995).

#### 2.3.3 Species tree inference

To estimate a species tree for our optimal species delimitation model, we used SNAPP with the Set 1 AFLP data. We employed default priors for mutation rates and ancestral population sizes, estimating both the forward and reverse mutation rates from the data. We used a gamma prior on the ancestral population sizes, with a shape parameter (α) of 11.75 and a scale parameter (β) of 109.73. We used a Yule prior for the species tree height and branch lengths with a lambda parameter of 0.00765. We ran two independent MCMC chains for 1 × 10^6^ generations, with parameters and trees sampled every 1,000 generations. To assess MCMC mixing and convergence, we visualized the output using Tracer v.1.6 (Rambaut, Suchard, Xie & Drummond, 2014). We summarized the posterior distribution of trees using TreeAnnotator v.2.1.2 (distributed with BEAST Bouckaert et al., 2014) with 25% burnin and mean node heights. We visualized the resulting maximum clade credibility tree using FigTree v. 1.4.2 (http://tree.bio.ed.ac.uk/software/figtree/).

### 2.4 Interactions between candidate species at areas of contact

Our delimitation of candidate *A. distichus* species was restricted to analyses of Set 1, which included broad geographic and taxonomic sampling. To test whether these candidate species are currently experiencing gene flow, we conducted separate analyses of Set 2 and Set 3, each of which contain samples from across contact zones between candidate species that have traditionally been recognized as subspecies.

For Set 2 and Set 3, we tested for the presence of distinct genotypic clusters corresponding with candidate species by conducting the same type of hierarchical STRUCTURE analyses used for the Set 1 analyses. By analyzing genotypic assignment proportions across the hybrid zone, we determined whether hybridization is ongoing, as well as the extent to which admixture is evident outside of the contact zone.

#### 2.4.1 Genetic diversity and pairwise $F_{\mathrm{ST}}$ calculation

We calculated genetic diversity ($H_{e}$) within and pairwise $F_{\mathrm{ST}}$ among each of the species identified in the optimal species delimitation model for Set 1 (see below) and for the genotypic clusters identified by independent STRUCTURE runs for Sets 2 and 3 using AFLP‐Surv v.1.0 (Vekemans, Beauwens, Lemaire & Roldan‐Ruiz, 2002). We ran 5,000 permutations of the Bayesian method with a nonuniform prior for allele frequencies (Zhivotovsky, 1999) to estimate $F_{\mathrm{ST}}$ under the assumption of Hardy–Weinberg equilibrium. For all three datasets, we calculated these statistics both with and without individuals from localities at known hybrid zones.

## 3 Results

### 3.1 Error rates and quality control

We determined AFLP scoring error rates for 20 locus/phenotype threshold combinations for each primer pair. Ultimately, we only used phenotype thresholds of >500 or greater because lower thresholds tended to result in noninterpretable results in downstream analyses, indicative of low‐quality data. Results were considered noninterpretable when STRUCTURE failed to assign more than 50% of the genomes of most individuals to any cluster. We were unable to determine primer pair specific error rates for sample Set 1 for two primer pairs (M53/E1 and M53/E2) due to a technical change at the core facility conducting our AFLP fragment analyses. As a result of this change, duplicate samples run with these two primer pairs had significantly higher AFLP peaks relative to the original samples, making comparison impossible. Thus, for all M53 primer pairs, we applied the filtering threshold most commonly used for all other primer pairs: a phenotype threshold of 500 and a locus threshold of 100.

Presence of an unusually low number of AFLP peaks resulted in complete exclusion of 10, 15, and 8 individuals from our three sample sets, resulting in 82, 77, and 51 retained individuals in Sets 1, 2, and 3, respectively. Due to low AFLP peaks, we excluded one primer pair from 7, 3, and 1 individual(s) and two primer pairs from 8, 1, and 0 individuals in sets 1, 2, and 3, respectively. Following exclusion of these primer pairs, the three sets were 97.6%, 99.2%, and 99.7% complete, respectively. The total number of loci retained was as follows: Set 1 included 534 loci, Set 2 included 552 loci, and Set 3 included 836 loci.

### 3.2 Species delimitation & species tree inference

#### 3.2.1 Genotypic clustering analyses

Set 1 contained 534 loci for 82 samples from across the range of *A. distichus*. The first round of Δ*K* analyses with Set 1 identified an optimal *K* of 2, with the two clusters largely corresponding with populations sampled from Hispaniola's North and South paleo‐islands (Figure S2a). This genotypic and geographic division occurs even within the only taxon that is broadly distributed on either side of Mertens’ line; individuals of *A. d. dominicensis* sampled from the North and South paleo‐island share genotypic assignments with other populations sampled from the same paleo‐island rather than with one another. The Bahamian subspecies of *A. distichus* and *A. altavalensis* had the majority of their genotypes assigned to the cluster associated Hispaniola's North paleo‐island.

The first hierarchical STRUCTURE analysis of the South paleo‐island cluster did not result in any further subdivision (lnP(D) greatest for *K* = 1, Figure S2a). The first hierarchical analyses of the North paleo‐island cluster suggested additional subdivision, with the optimal *K* = 2. However, the two genotypic clusters identified by this analysis were not easily interpretable, with many individuals from the same subspecies and locality assigned to different clusters. Additionally, most individuals’ genotypes were not strongly assigned to any one cluster (average of maximum genotype assignment proportions = 58.5%).

Closer inspection of other values of *K* from the hierarchical analysis of North paleo‐island cluster revealed a more readily interpretable pattern at *K* = 4, the value of *K* with the highest overall lnP(D). For *K* = 4, most individuals had the majority of their genotype assigned to a single cluster (average of maximum genotype assignment proportions = 83.8%, Figure S2a). The four genotypic clusters identified in analyses of North Paleo‐island populations corresponded primarily with the following populations: (1) *A. d. ravitergum*, individuals from a hybrid zone between *A. d. ravitergum* and *A. d. ignigularis*,* A. altavalensis*, and *A. d. dominicensis* from the central Dominican Republic, (2) *A. d. ignigularis* and individuals from a hybrid zone between *A. d. ravitergum* and *A. d. ignigularis*, (3) *A. d. properus* and *A. d. sejunctus*, and (4) the Bahamian subspecies and individuals of *A. d. dominicensis* from the north‐central Dominican Republic. Only two individuals did not have the majority of their genotype assigned to a single genetic cluster: one *A. d. dominicensis* from the central Dominican Republic and one individual from the hybrid zone between *A. d. ravitergum* and *A. d. ignigularis*.

After recovering a total of five genotypic clusters (one in the South paleo‐island and four in the North paleo‐island) via hierarchical analyses, we ran 100 replicate STRUCTURE runs on Set 1 with *K* fixed at 5. However, this final analysis produced slightly different clustering than was suggested by the hierarchical analyses (Figures 1 and S2b). These differences included (1) the nature of genotypic division within *A. d. dominicensis*, (2) the tendency for more Dominican subspecies to share a genotypic cluster with the Bahamian distichoids, (3) identification of a genotypic cluster associated with *A. d. favillarum* rather than grouping this subspecies with other South paleo‐island populations, and (4) lumping of *A. d. properus* and *A. d. sejunctus* with *A. d. ignigularis*. We evaluated the fit of the these two clustering schemes separately (models VI and VII) and in combination (model VIII) using BFD*.

#### 3.2.2 Bayes factor delimitation

We compared eight different species delimitation scenarios under a coalescent‐based framework (Figure 2). Model VIII had the largest marginal likelihood, so all Bayes factors were calculated relative to this model. Model VIII was based on the five genotypic clusters identified by the final STRUCTURE analysis with 100 replicates fixed at *K* = 5, plus a sixth cluster of *A. d. properus/sejunctus* identified by the hierarchical STRUCTURE analyses. Bayes factors were >10 for all pairwise comparisons, indicating decisive support for model VIII as the optimal species delimitation model.

Model VIII is composed of six species. The first candidate species is a South paleo‐island endemic that includes all populations of the highly polymorphic *A. d. favillarum*. The second candidate species is also largely endemic to the South paleo‐island and includes all populations of the Tiburon Peninsula endemic subspecies (*A. d. aurifer*,* A. d. suppar*, and *A. d. vinosus*) as well as southern populations of the widespread *A. d. dominicensis*. The northern boundary for this candidate species is ambiguous because it abuts the range of another candidate species containing northern populations of phenotypically similar *A. d. dominicensis*. Like the first candidate species, this second candidate species also includes extensive variation in dewlap color and pattern, with dewlaps that range from wine red (e.g., *A. d. vinosus*) to pale yellow (e.g., *A. d. suppar*).

The third candidate species is found primarily on the North paleo‐island and includes all populations of *A. d. ravitergum* as well as the satellite island endemic *A. altavelensis*. The range of this candidate species is disjunct, as it includes populations from both the south‐central Dominican Republic and the island of Alto Velo off the southern coast of the Barahona Peninsula. Both dewlap and body color are highly polymorphic in this candidate species, with *A. d. ravitergum* tending to have gray or pale brown bodies and pale yellow dewlaps whereas *A. altavelensis* have striking orange bodies and dewlaps.

The fourth candidate species is a North paleo‐island endemic that includes all populations of *A. d. ignigularis*. The range of this candidate species encompasses the central and eastern Dominican Republic. This candidate species exhibits some variation in body and dewlap coloration, but most populations have largely green dorsal body coloration and dewlaps with a substantial amount of orange.

The fifth candidate species is another North paleo‐island endemic that includes *A. d. properus* from the western Dominican Republic and *A. d. sejunctus* from Isla Saona, a nearby satellite island. Representatives of this candidate species also exhibit considerable variation in dewlap color and pattern.

The sixth candidate species includes northern *A. d. dominicensis* and the two Bahamian island subspecies (*A. d. distichus* and *A. d. ocior*). All of the populations assigned to this candidate species tend to have relatively pale dewlaps. One of the Bahamian subspecies included in this group (*A. d. ocior*) has the most green body coloration of any distichoid population (Schwartz, 1968).

#### 3.2.3 Species tree inference

We used the BEAST package SNAPP to infer phylogenetic relationships among the candidate species identified by model VIII (Figure 4). All parameters achieved ESS values >500 after 2 × 10^6^ MCMC generations, and both independent runs converged on similar posterior distributions. In the resulting tree, the South paleo‐island populations of *A. distichus* formed a monophyletic group with moderate support (posterior probability = 0.82). However, the strongly supported placement of the predominantly North paleo‐island populations of *A. d. dominicensis* (Figure 4, Species F) rendered the North paleo‐island paraphyletic. We also observed a weakly supported sister relationship between the *A. d. ignigularis* species and the *A. d. properus/sejunctus* species.

#### 3.2.4 Population structure statistics for candidate species

Pairwise $F_{\mathrm{ST}}$ and $H_{e}$ values for Set 1 are reported in Table 2. Pairwise $F_{\mathrm{ST}}$ values were slightly higher when individuals from the hybrid zone between *A. d. ravitergum* and *A. d. ignigularis* were excluded. The largest pairwise $F_{\mathrm{ST}}$ values were observed between the *A. d. ravitergum/altavalensis* group and the two South paleo‐island groups. Overall, low $F_{\mathrm{ST}}$ values indicate gene flow may still be ongoing between all of candidate species.

**Table 2:** $H_{e}$ and pairwise $F_{\mathrm{ST}}$ values for the six species of delimitation model VIII. Values shown were calculated without including individuals from the hybrid zone between *A. d. ravitergum* and *A. d. ignigularis*

	Pairwise $F_{\mathrm{ST}}$						
	$H_{e}$	*A. d. favillarum*	*A. d. ignigularis*	*A. d. ocior*/*distichus*/*dominicensis* N	*A. d. suppar*/*aurifer/vinosus*/*dominicensis* S	*A. d. ravitergum*/*altavalensis*	*A. d. properus*/*sejunctus*
*A. d. favillarum*	0.01937	0					
*A. d. ignigularis*	0.03367	0.1706	0				
*A. d. ocior/distichus/dominicensis* N	0.03408	0.1170	0.0895	0			
*A. d. suppar/aurifer/vinosus/dominicensis* S	0.02206	0.1415	0.1651	0.0683	0		
*A. d. ravitergum/altavalensis*	0.03458	0.2851	0.1193	0.1660	0.2704	0	
*A. d. properus/sejunctus*	0.03425	0.1587	0.0142	0.1032	0.1662	0.1322	0

### 3.3 Interactions between candidate species at areas of contact

**Figure 3:**
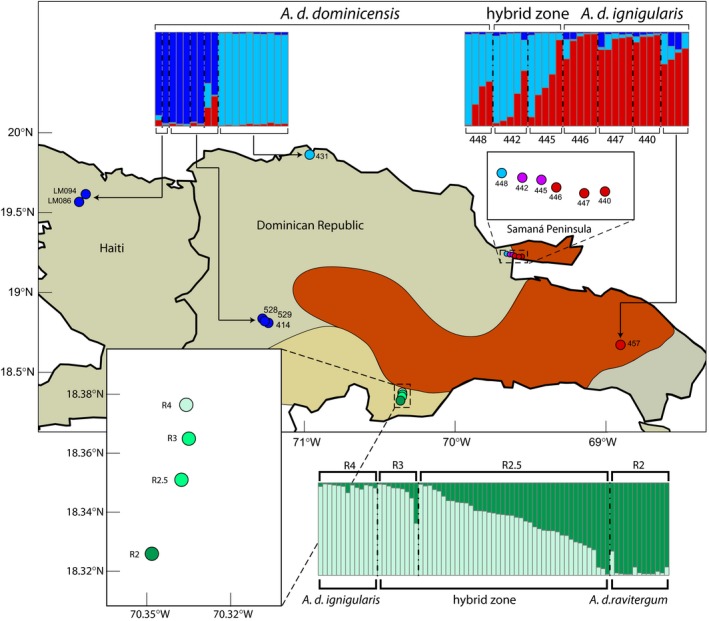
Sampling and results from independent genotypic clustering analyses conducted in STRUCTURE for Set 2 (green bar plots) and Set 3 (blue and red bar plots). Each column on the bar plots represents an individual sample. Different colors correspond to different genetic clusters. Shading of each column represents the proportion of the genome for that individual assigned to one of the genetic clusters identified by STRUCTURE. Each point on the map is a locality included in Set 2 or Set 3, labeled with corresponding locality numbers. The color of each locality reflects the genetic cluster to which the majority of the genomes at that locality were assigned. The purple coloring of localities 442 and 445 and the bright green coloring of localities R2.5 and R3 represent their admixed status

Our second set of samples consisted of 552 AFLP loci for 77 *A. d. ravitergum* and *A. d. ignigularis* from a transect that spans a zone of contact between the two subspecies. The first round of Δ*K* analyses with this dataset identified an optimal *K* = 2, with clusters corresponding largely with subspecies (Figure 3). No further population structure was revealed with additional hierarchical Δ*K* analyses. The genotypes of all *A. d. ignigularis* individuals from the northern end of the transect were strongly assigned (min = 89.3%, mean = 96.0%) to one genotypic cluster. Genotypes of the all *A. d. ravitergum* individuals from the southern end of the transect were strongly assigned (min = 73.9%, mean = 95.0%) to the second genotypic cluster. Individuals from sites in the middle of the transect were admixed, with genotypes assigned to both clusters. The two sites in the middle of the transect were very heterogeneous, with genotype assignment proportions for the (*A. d. ravitergum*) cluster ranging from 1.4% to 98.8%.

Our third set of samples consisted of 836 AFLP loci for 51 individuals sampled across the ranges of *A. d. ignigularis* and *A. d. dominicensis*, with a particular focus on a transect between these two subspecies. The first round of Δ*K* analyses with this dataset identified *K* = 3 as optimal. Subsequent hierarchical Δ*K* analyses failed to recover any additional population structure. *A. d. dominicensis* individuals from Haiti and the central Dominican Republic had the majority (min = 54.4%, mean = 87.9%) of their genotypes assigned to one cluster. The remaining *A. d. dominicensis* individuals from the northeastern Dominican Republic and the western edge of the transect had the majority (min = 55.0%, mean = 88.7%) of their genotypes assigned to a second cluster. All *A. d. ignigularis* individuals from the eastern edge of the transect and the southeastern Dominican Republic had the majority (min = 66.0%, mean = 87.4%) of their genotypes assigned to a third cluster. Individuals from two sites in the middle of the transect (Figure 3, localities 442 and 445) had admixed genotypes assigned primarily to the later two clusters. In the middle of the transect, the proportion of genotypes assigned to the third, predominantly *A. d. ignigularis* cluster ranged from 3.2% to 91.7%.

We also calculated pairwise $F_{\mathrm{ST}}$ and $H_{e}$ values for the clusters identified by independent STRUCTURE analyses of sets 2 and 3. Pairwise $F_{\mathrm{ST}}$ estimates between *A. d. ravitergum* and *A. d. ignigularis* for Set 2 were larger when individuals from the hybrid zone were excluded (0.1071 vs. 0.2536). Pairwise $F_{\mathrm{ST}}$ estimate was also larger for Set 3 when excluding potential hybrids (0.0922 vs. 0.1152 between *A. d. ignigularis* and northeastern *A. d. dominicensis*, 0.1151 vs. 0.1163 between northeastern *A. d. dominicensis* and central Dominican/Haitian *A. d. dominicensis*, and 0.1502 vs. 0.1527 between *A. d. ignigularis* and central Dominican/Haitian *A. d. dominicensis*).

## 4 Discussion

Using genotypic clustering and species delimitation methods, we recover strong support for the hypothesis that *A. distichus* is comprised of numerous genomically distinct populations, likely representing independently evolving evolutionary lineages that warrant recognition as distinct species under the general lineage concept. Although some of the putative species identified by our analyses closely correspond with previously diagnosed subspecific boundaries (*A. d. favillarum*,* A. d. ignigularis*), most do not (Figure 2). Lack of correspondence between genomically distinct populations and subspecific boundaries is due both to the fact that some subspecies with divergent dewlap color are inferred to share similar genomes (e.g., the three subspecies endemic to the Tiburon Peninsula) and the fact that populations from one widespread subspecies (*A. d. dominicensis*) are inferred to include numerous genomically distinct populations. While these results do not support the hypothesis that dewlap color and pattern variation is necessarily associated with divergence of distinct species, they do support the hypothesis that geographic isolation has likely played an important role in driving divergence across populations of bark anoles. Genetic structure is largely congruent with the division between the North and South paleo‐islands of Hispaniola. Our results also indicate fairly recent colonization of Hispaniola's satellite islands by mainland Hispaniolan anoles.

**Figure 4:**
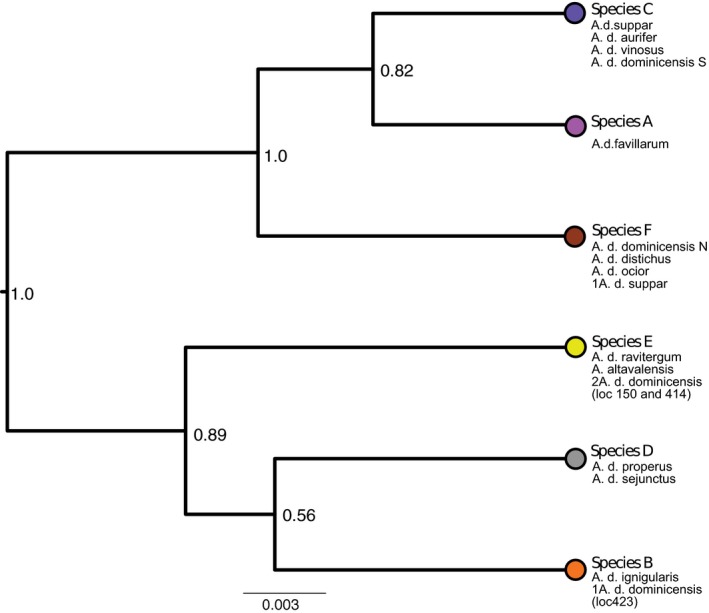
Species tree inferred for the candidate species in model VIII. Nodes are labeled with Bayesian posterior probability values. Tips are labeled with candidate species names and the subspecies contained within. Localities for the *A. d. dominicensis* in species B and E are noted. Tip colors correspond to the genetic cluster colors in Figure 1, with the exception of species D which in Figure 1 is part of the orange cluster containing *A. d. ignigularis*

Assessment of alternative species delimitation scenarios with AFLP genome scan data strongly support a scenario derived from genotypic clustering analyses that divides the *A. distichus* species group into six candidate species (Figures 2 and 4). Our species delimitation analyses included a few individuals from a hybrid zone between *A. d. ignigularis* and *A. d. ravitergum*. We chose not to exclude these admixed individuals as this may have falsely inflated support for delimitation models comprising more species. However, despite the inclusion of these admixed individuals, the optimal species delimitation model still split *A. d. ravitergum* and *A. d. ignigularis* into different candidate species. The inclusion of admixed *A. d. ignigularis/ravitergum* individuals in our species tree analysis may explain the low posterior probability for the split between the *A. d. properus/sejunctus* candidate species and the primarily *A. d. ignigularis* candidate species (Figure 4).

One caveat to our delimitation of *A. distichus* is that nearly all of our estimates of pairwise $F_{\mathrm{ST}}$ values are lower than those reported by another study using similar population structure analyses of AFLPs in the clade sister to *A. distichus* (Lambert et al., 2013). In that study, the smallest interspecific pairwise $F_{\mathrm{ST}}$ value (0.3357) was greater than the largest $F_{\mathrm{ST}}$ value we observed among *A. distichus* populations (0.2851, between *A. d. ravitergum/alavalensis* and *A. d. favillarum*) (Table 2) (Lambert et al., 2013). This observation supports the hypothesis that divergence within populations currently recognized as *A. distichus* is younger than that observed between the four distinct species previously recognized as *A. brevirostris* (Arnold, 1980).

### 4.1 Biogeography

Although pure biogeographic scenarios were among the worst performing delimitation models (Figure 2), our results support prior hypotheses (Geneva et al., 2015; Glor & Laport, 2012) that suggest divergence of populations on Hispaniola's North and South paleo‐islands has contributed to diversification in bark anoles (Figures 1 and S2). The first division in our hierarchical STRUCTURE analyses distinguishes populations found primarily on Hispaniola's North and South paleo‐islands. Our analyses are unable to determine whether this divergence across the paleo‐island boundary occurred prior to the paleo‐island merger or from restricted gene flow since the merger due periodic inundation of the paleo‐island boundary or the inhospitable environmental conditions of this region (Glor & Warren, 2011).

Our study also sheds light on the origin of *A. distichus* populations that are not found on mainland Hispaniola. The fact that the Bahamian populations (*A. d. distichus* and *A. d. ocior*) are genomically indistinguishable from populations of *A. d. dominicensis* found in northern Hispaniola supports Geneva et al.'s (2015) hypothesis that the Bahamian populations are the result of relatively recent overwater dispersal. Increased taxonomic coverage and geographic sampling of the Bahamanian subspecies and *A. d. dominicensis* will be crucial to pinpoint the progenitor population(s) of the Bahamian distichoids and to determine when the Bahamas were colonized. We also find evidence for recent colonization of Isla Saona by *A. d. sejunctus*, which is only weakly phenotypically and genetically differentiated from *A. d. properus*, the closest mainland subspecies. Finally, we find support for the hypothesis from Geneva et al. (2015) that *A. altavalensis*, which is endemic to the southernmost satellite island of the Dominican Republic (Isla Alto Velo), likely resulted from relatively recent colonization of this island by *A. d. ravitergum*. There are no *A. distichus* on mainland Hispaniola in the arid and potentially inhospitable Barahona Peninsula adjacent to Isla Alto Velo. Our results suggests that *A. distichus* colonized Isla Alto Velo either when the species was previously distributed in closer proximity to this island or via long‐distance over‐water dispersal of at least 100 km from the current range of *A. d. ravitergum*.

### 4.2 Dewlap color in species delimitation

The historic use of dewlap color as the primary taxonomic character in the *A. distichus* complex has led to recognition of many subspecies that may not reflect true evolutionary lineages. We identified several candidate species that contain a broad array of dewlap colors. For instance, *A. d. favillarum* appears to be a single genetic population with impressive dewlap color polymorphism, consistent with prior phylogenetic (Geneva et al., 2015) and allozyme studies (Case, 1990; Williams & Case, 1986). In another case of dewlap polymorphism without genetic divergence, four parapatric *A. distichus* subspecies on the Tiburon Peninsula of Southwestern Haiti, *A. d. aurifer*,* A. d. suppar*,* A. d. vinosus*, and *A. d. dominicensis* each have distinct dewlap coloration, yet make up a single genetic cluster (Figure 1). This “genetic continuity” of the three Tiburon subspecies was previously hinted at by the unfinished allozyme work of Webster in the 1970s (Williams, 1977). On the other hand, at least one previously delimited subspecies with similar dewlap color across its range appears to represent multiple independent evolutionary lineages; populations of *A. d. dominicensis* were split across four separate candidate species, in agreement with prior phylogenetic results (Geneva et al., 2015). Together these results suggest that dewlap color is not by itself a reliable diagnostic trait in the *A. distichus* complex, and perhaps in anoles more broadly. Other polymorphic anoles may also be composed of multiple genetically divergent species, which implies that the biodiversity of anoles is currently underestimated. Future studies should explicitly quantify both dewlap color variation and genetic variation to determine whether other anole species exhibit a similar disassociation between dewlap color and population structure (e.g. Ng et al., 2016).

### 4.3 Hybridization and introgression

We examined two *A. distichus* subspecies pairs for evidence of hybridization at contact zones. *A. d. ignigularis* (Figure 4, candidate species B) and *A. d. ravitergum* (Figure 4, part of candidate species A) come into contact in the southern Dominican Republic along the Baní River. This contact zone, first described by Williams (1977) appears to be facilitated by the intrusion of mesic habitat, characteristic of *A. d. ignigularis*, into otherwise xeric habitat, home to *A. d. ravitergum*. Our transect follows a road along the Baní River, transitioning from xeric habitat in the south to mesic habitat in the north (Ng et al., 2016). Our genotypic clustering analyses reveal a strong signal of admixture in the middle of this transect with very little admixture at either end (Figure 3). This pattern is indicative of hybridization without substantial gene flow into the home range of either subspecies (Ng et al., 2016). Despite low pairwise $F_{\mathrm{ST}}$ estimates between the two subspecies, we conclude that there is a strong genetic break between *A. d. ignigularis* and *A. d. ravitergum*, with admixture at the hybrid zone but limited gene flow between the subspecies.

The second subspecies pair we examined was *A. d. ignigularis* (Figure 3, candidate species B) and *A. d. dominicensis* (divided among four candidate species). Our transect for these subspecies runs east–west, spanning a recently recessed marine channel that separated the Samaná Peninsula from mainland Hispaniola (Grant, 1956; Ng et al., 2016). Unlike the transect between *A. d. ravitergum* and *A. d. ignigularis*, this transect does not encompass any obvious environmental gradient. While there is signal of admixture in the middle of this transect, the hybrid zone is not as well defined as the hybrid zone between *A. d. ravitergum* and *A. d. ignigularis* (Ng et al., 2016). There appears to be significant admixture well into the range of *A. d. dominicensis* at the western edge of the transect. Thus, we conclude that *A. d. ignigularis* genetic material has effectively introgressed into *A. d. dominicensis* beyond the contact zone. However, without further geographic sampling of *A. d. dominicensis* populations in eastern Hispaniola, it is difficult to determine the extent of this gene flow.

Previous phylogenetic analyses found that *A. d. dominicensis* consists of three or four geographically distinct and deeply divergent polyphyletic lineages (Geneva et al., 2015). Their species tree analyses recovered a clade of northern Haitian/central Dominican *A. d. dominicensis* and a separate clade of northern Dominican *A. d. dominicensis* whose most recent common ancestor was that of all *A. distichus* (Geneva et al., 2015). This deep divergence within *A. d. dominicensis* is reflected in our own analyses, with a distinct genetic break between populations from northern Haiti/central Dominican Republic and populations from the northeastern Dominican Republic (Figure 3). Comparatively, *A. d. ignigularis* located on mainland Hispaniola shows little genetic differentiation across its entire range, from the Samaná Peninsula to the southeastern Dominican Republic. Thus, despite the relative uniformity of dewlap color, there is genetic evidence for multiple independent lineages within the widespread *A. d. dominicensis*.

## 5 Taxonomic Recommendations

Our results together with prior work strongly suggest that formal taxonomic revision of populations previously recognized as *A. distichus* is needed because this species is comprised of numerous distinct populations that likely warrant recognition as distinct species. We have not undertaken such a taxonomic revision here because we are unable to provide diagnostic phenotypic traits to distinguish the candidate species identified on the basis of genomic differentiation. Additionally, delimiting the geographic boundaries between these putative species requires more extensive geographic sampling of genomic variation. The fact that *A. altavalensis* is genetically indistinguishable from *A. d. ravitergum* could be used to argue in favor of no longer recognizing the Alto Velo populations of bark anoles as a distinct species. However, we agree with the suggestion by Geneva et al. (2015) that *A. altavalensis* warrants continued recognition because it is clearly geographically isolated and phenotypical distinct from *A. d. ravitergum*.

## 6 Conclusions

Our study provides a geographically broad first‐take genomic perspective on a young species complex of anoles with remarkable dewlap color polymorphism. Consistent with results from mitochondrial DNA (Geneva et al., 2015; Glor & Laport, 2012) and several nuclear genes (Geneva et al., 2015), we find strong evidence for genetic differentiation despite some gene flow between the lineages of *Anolis distichus*. We identify six new candidate species with our molecular species delimitation and suggest that *A. altavalensis* should be maintained as a seventh species. The genetic breaks and candidate species we recovered are largely unassociated with shifts in dewlap coloration. We conclude that dewlap color is a highly labile trait that may be misleading if used as the primary diagnostic character for species delimitation. Thus, there is likely substantial unrecognized biodiversity within other polymorphic anole species.

In contrast to the lack of genetic divergence between populations differing in dewlap coloration, we find support for several biogeographic hypotheses. First, we find evidence for a genetic break between populations of *A. distichus* on the North and South paleo‐islands of Hispaniola. We also observe that the Hispaniola satellite island endemic *A. d. sejunctus* appears to be the result of colonization by the nearest mainland subspecies, *A. d. properus*, suggesting reconsideration of satellite island endemics as distinct subspecies. In an example of long‐distance dispersal to a satellite island, *A. altavalensis* was likely founded by *A. d. ravitergum* traveling at least 100 km over‐water. We also posit that the Bahamian distichoids are the result of colonization by *A. d. dominicensis* from northern Hispaniola. Our insight into such biogeographic patterns will only grow clearer as future studies increase in genomic, taxonomic, and geographic scope.

## Conflict of Interest

None declared.
