# Supplementary figures and images for: A genomic assessment of species boundaries and hybridization in a group of highly polymorphic anoles (distichus species complex)
Source: Ecol Evol. 2017 Apr 15;7(11):3657–71. doi: 10.1002/ece3.2751 (PMC5468153; doi:10.1002/ece3.2751)

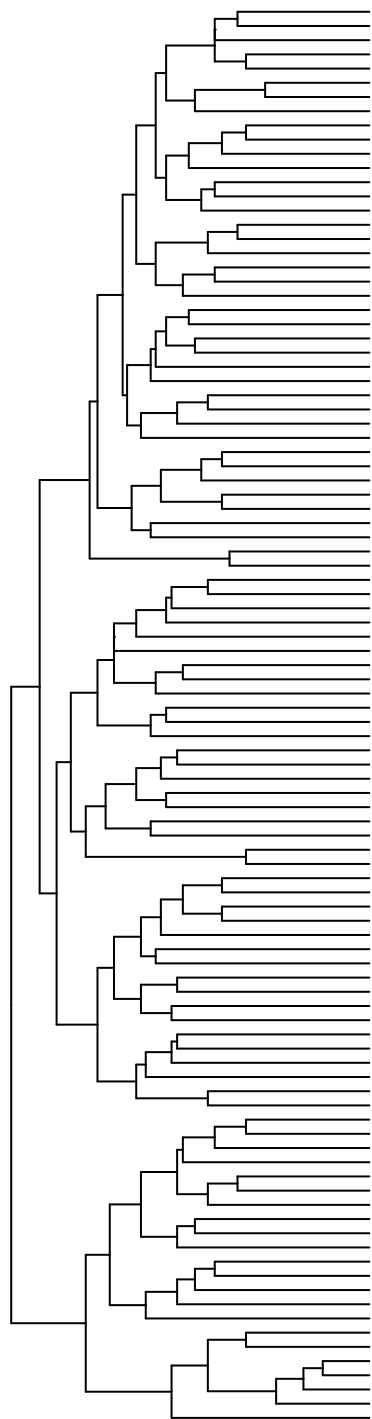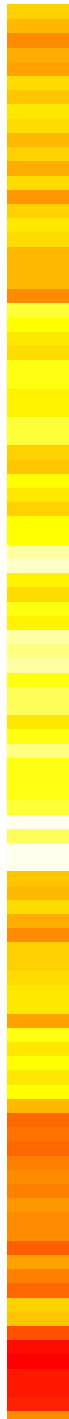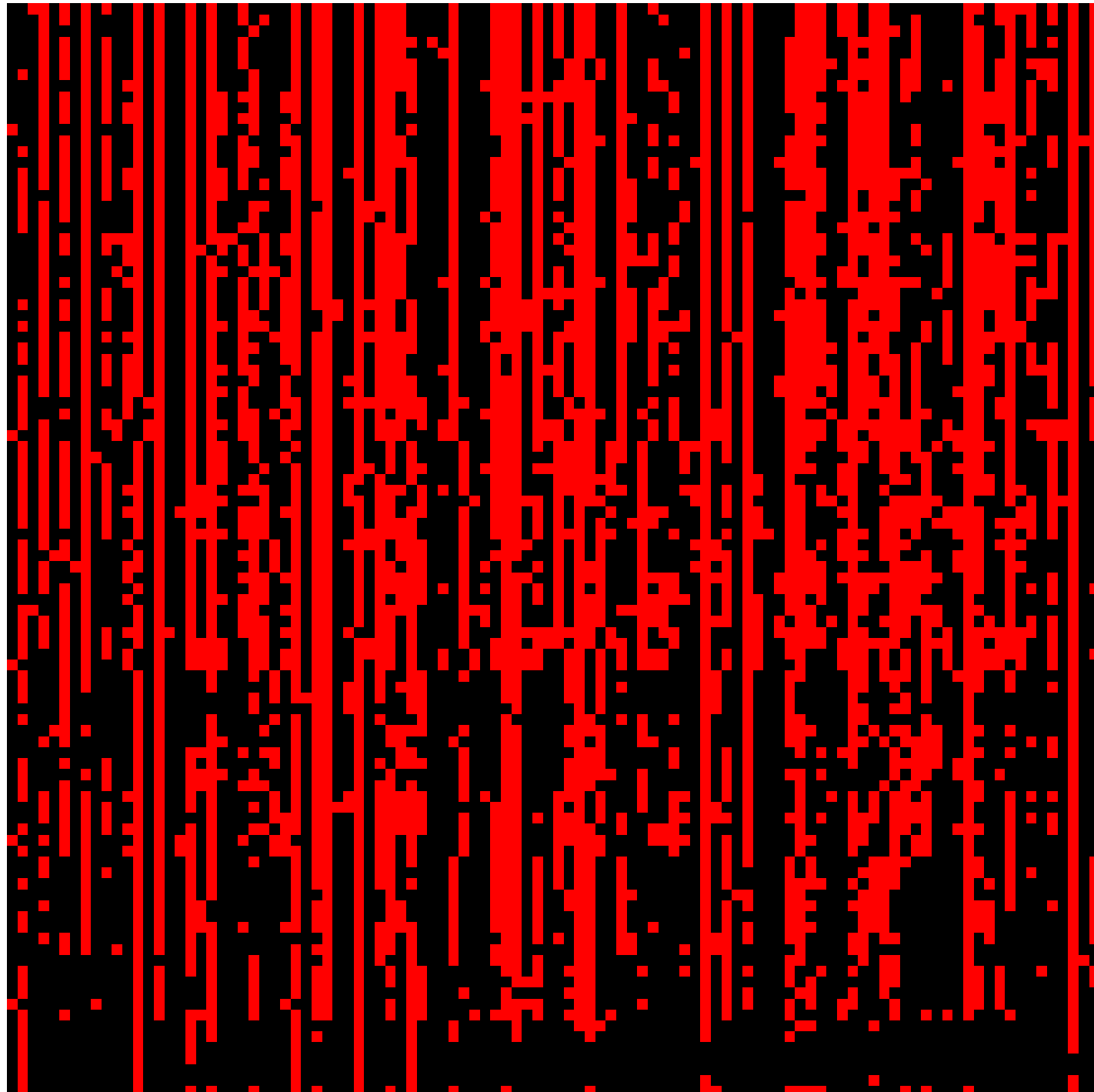

8288  
8287  
8289  
8304  
8290  
8285-dup  
8286  
7438-dup  
7441-dup  
7440-dup  
8289-M51  
8307  
8311  
8313  
5772-dup  
5777-dup  
5142  
8184  
8179  
4375  
8308  
8337-dup  
8328  
8303  
8291  
8329  
8187  
8186  
8185  
8178  
8305  
8312  
8306  
8309  
8309-M51  
8284  
4326  
8302  
8302-M51  
3776  
3776-M51  
6571  
6575  
6460-dup  
6371  
6435-dup  
6436-dup  
6452-dup  
266170  
7509  
1264  
8485  
7612  
8482  
4433  
6406  
6457-dup  
6504  
6454-dup  
6454-dup-M51  
266168  
266169  
266166  
266167  
5095  
8483  
4875  
8484  
8498  
5081  
4691  
7599  
7619  
7618  
7611  
7613-M51  
7613  
3814-dup  
5779-dup  
3813-dup  
8182  
8337  
8338  
8338-M51  
8183  
8180  
8181  
192880  
192881  
192920  
192881-M51  
5078  
5169  
5691  
5170  
5176  
5174  
5690  
4846

Supplement: Supplementary file 1 [file ECE3-7-3657-s001.pdf]

A

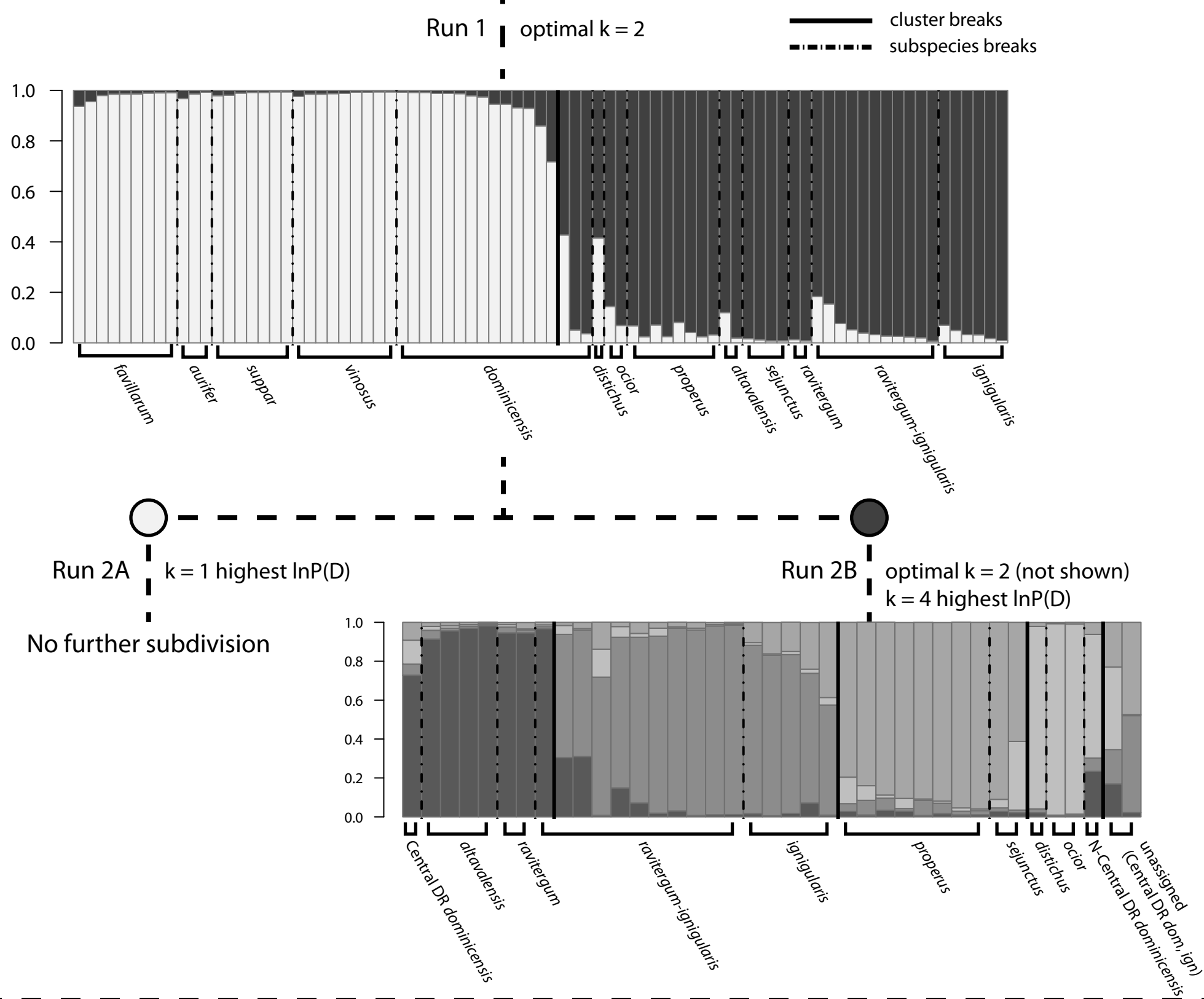

B

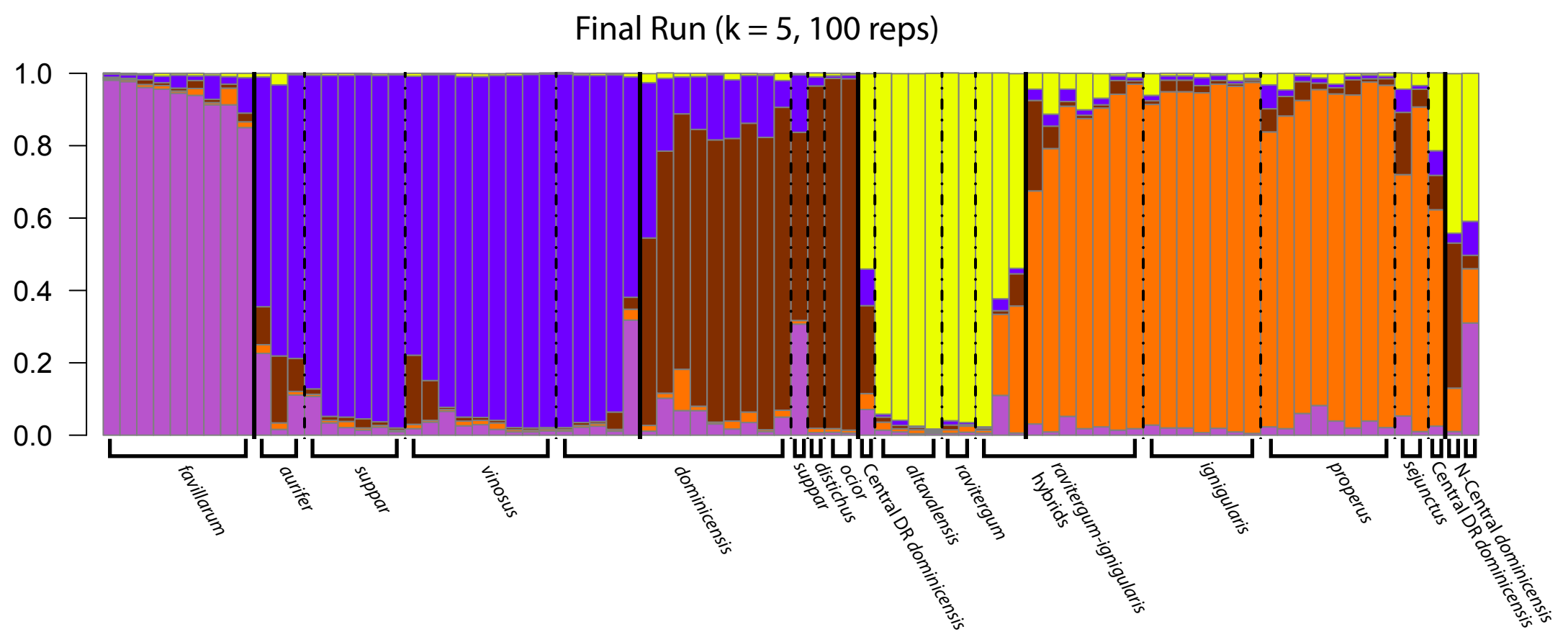

Supplement: Supplementary file 2 [file ECE3-7-3657-s002.pdf]
